# Navigating the Healthcare System with a Complex Chronic Condition: Multidimensional Roles of Adolescents, Young Adults, and Parents

**DOI:** 10.3390/children12030318

**Published:** 2025-02-28

**Authors:** Allison F. Wise, Emily J. Upham, Danielle D. DeCourcey

**Affiliations:** 1 Department of Supportive Oncology, Dana Farber Cancer Institute, Boston, MA 02215, USA; 2 Division of Medical Critical Care, Department of Pediatrics, Boston Children’s Hospital, Boston, MA 02115, USA; emily.upham@childrens.harvard.edu (E.J.U.); danielle.decourcey@childrens.harvard.edu (D.D.D.)

**Keywords:** pediatric palliative care, decision-making, qualitative research, life-limiting illness, life-threatening illness, serious illness, healthcare transition

## Abstract

Background/Objective: Adolescents and young adults with complex chronic conditions and parents of children with complex chronic conditions interact frequently with the healthcare system. However, these groups have unique characteristics and needs. The objective of this qualitative study was to explore and compare the roles that adolescents and young adults with complex chronic conditions and parents of children with complex chronic conditions take on when engaging with the healthcare system. Methods: Semi-structured interviews were conducted with seven adolescents and young adults and nine parents at two pediatric centers from December 2018 to April 2019. The research team conducted thematic analysis. Transcripts were coded independently by two coders, achieving high interrater reliability (kappa > 0.85). We present findings related to illness experience and self-described roles within the healthcare context. Results: Informational roles described by both parents and adolescents and young adults included teacher, learner and researcher, and planner. Social-emotional roles common to both groups included source of strength, support, and hope and worrier, while the role of guardian was unique to parents. Action-oriented roles described by both groups included advocate, decision-maker, and communicator, while medical care provider was unique to parents and medical care manager was unique to adolescents and young adults. Conclusions: Adolescents and young adults with complex chronic conditions and parents of children with complex chronic conditions balance similarly complex roles within the medical system. However, their experiences within these roles can differ, presenting unique challenges. Understanding these multidimensional roles will better equip healthcare providers to support these patients and families, especially around times of healthcare transition.

## 1. Introduction

As medical technologies continue to advance, more children are living with serious, complex, and chronic illnesses [1,2]. Adolescents and young adults (AYAs) with complex chronic conditions (CCCs) and parents of children with CCCs face a challenging healthcare system in the United States that is fragmented, uncoordinated, and poorly designed to meet the needs of children with medical complexity [3]. To navigate this system, both groups take on a complex interplay of roles to ensure they receive the best medical care. Parents of children with medical complexity report losing their parent identity as they become decision-makers, detectives, advocates, guards, case managers, students, educators and healthcare providers [4,5,6,7,8].

For AYAs with CCCs, transitioning to adulthood means assuming responsibilities previously held by their parent or guardian [9,10]. Many AYAs take on the role of managing their own medical care, though the level of shared responsibility with a parent or caregiver evolves uniquely with an AYA’s developmental and illness trajectory [11,12]. Research has shown that AYAs with cancer, for example, often develop strong and specific preferences regarding the division of responsibility in decision-making. In one study, 58% desired sharing decision-making responsibilities with their oncologist, and 51% preferred limited parental involvement [13]. Similarly, AYAs with chronic diseases such as juvenile rheumatoid arthritis, sickle cell disease, inflammatory bowel disease, and kidney disease preferred direct communication with providers and to be actively involved in decisions about their care [14,15]. A recent study of AYAs with cystic fibrosis described their transition into the role of advocate, although some parents reassumed this role in “critical moments” of the illness trajectory [16].

Most research characterizing the responsibilities of AYAs with CCCs has focused on the roles of medical care manager, decision-maker, and communicator, but less attention has been given to other roles they may take on, or how they evolve over time. Additionally, much of the existing literature has focused on AYAs with advanced cancer [13,14,15,17], or narrowly focuses on individual disease populations. Few studies have explored the breadth of roles adopted by AYAs navigating diverse CCCs.

Recognizing this gap in the literature, the objective of this qualitative study was to explore the experiences of AYAs with CCCs and parents of children with CCCs to characterize the broad range of roles each group takes on when engaging with the healthcare system. We also aimed to identify similarities and differences between the two groups. The goal of this work is to inform the development of tailored supports and services for these groups, who interact with the healthcare system frequently and at the most vulnerable moments in their lives.

## 2. Materials and Methods

This study was a sub-analysis of data from the Pediatric Serious Illness Communication Program study, which developed a structured approach to advance care planning discussions for children/AYAs with CCCs and their parents [18]. As part of program development, semi-structured interviews were conducted with parents and seriously ill AYAs at Boston Children’s Hospital (BCH) and Dana–Farber Cancer Institute from December 2018 to April 2019. Interviews explored perceptions about living with a serious illness, current serious illness care, and communication. This study employed purposive sampling based on illness type, duration of illness, and gender to maximize variation. Eligible AYAs were those with a complex chronic condition [19], aged 13–35, English-speaking, and identified by their attending physician to be cognitively able to participate. Eligible parents included English-speaking parents of any age child living with a CCC. Potential patient and parent participants were either self-referred after viewing study flyers in outpatient or inpatient settings or on the Courageous Parents Network website (www.courageousparentsnetwork.org), or they were referred by the pediatric palliative care or critical care service. Participants not self-referred were approached in person, following attending/primary physician approval, and invited to participate.

Through an iterative process, the research team developed distinct interview guides for AYAs and parents, with input from a diverse 16-member national expert panel of clinical researchers, multidisciplinary healthcare professionals, and parents from the BCH Patient and Parent Advisory Council and the Courageous Parents Network [20]. Interview guides focused on communication with medical providers, experiences during unplanned hospital admissions, current illness understanding, and advance care planning. Semi-structured interviews were conducted by a trained interviewer and lasted approximately 60 min.

Two researchers (AW, EU) with expertise in qualitative methods conducted thematic analysis on transcripts from 15 interviews. Comprehensive coding structures were iteratively developed for both parents and AYAs, incorporating prefigured and emergent codes [21,22,23]. Text was examined for themes related to self-described roles within the medical system. The coding structures were then systematically applied and coded independently by the team, achieving high inter-rater reliability (kappa > 0.85) [24]. Discrepancies were identified and discussed weekly by the research team until a high level of agreement was achieved with adjudication by a third qualitative researcher (DD) when necessary. Thematic analysis prioritized the identification of key concepts, contexts, and patterns to understand predominant themes. Data were stored and organized using NVivo12 data management software (QSR International, Doncaster, Australia) [25]. Methodologic rigor was established through prolonged engagement and peer debriefing following the Consolidated Criteria for Reporting Qualitative Research (COReQ) [26].

## 3. Results

A total of nine parents and seven AYAs completed interviews, including one parent–AYA dyad. Table 1 shows participant characteristics. The mean parent age was 52 years, and those parents’ children had a mean age of 18 years; the mean AYA age was 24 years. The majority of participants in both groups were female (67% parents, 81% AYAs) and white (89% parents, 86% AYAs). A wide variety of disease categories were represented by both participant groups, including congenital and chromosomal, cardiac, oncologic, and pulmonary. No children of parent participants had renal disease; no AYAs had either static or progressive encephalopathies. The pediatric palliative care team was involved in patient care for over 70% of both groups’ participants.

**Figure 1:**
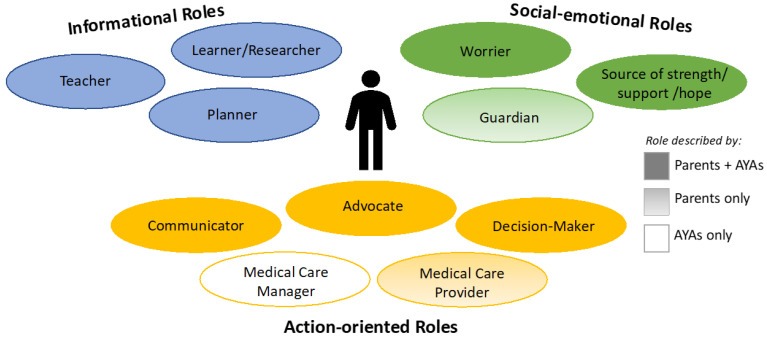
Schematic of roles described by parents of children with CCCs and AYAs with CCCs. Ovals filled with solid colors indicate roles described by both parents and AYAs. Ovals filled with gradient shading indicate roles described by parents only, and those with no shading indicate roles described by AYAs only.

Self-identified roles fell into three primary categories for both parents and AYAs, depicted in Figure 1: informational roles, social-emotional roles, and action-oriented roles. Informational roles included teacher, learner and researcher, and planner. Social-emotional roles included worrier; source of strength, support, and hope; and guardian. Action-oriented roles included advocate, decision-maker, communicator, medical care provider, and medical care manager. Most roles applied to both parents and AYAs. The role of guardian (social-emotional) and medical care provider (action-oriented) were described by only parents. The role of medical care manager (action-oriented) was described by only AYAs. Each category of roles is elucidated below with specific exemplar quotes detailed in Table 2, Table 3 and Table 4.

- Informational roles (Table 2):

***Teacher*:** Parents described the role of educating medical care providers about their child’s medical history, including prior diagnoses, medications, and how their child responded to prior treatments. Parents described the importance of sharing this personal knowledge about their child’s baseline, including who they are as a person, with new members of their child’s care team. As one parent explained,

“*… everybody here, you’re all the expert at the medicine part, and we’re the expert at the [NAME] part*”.

(Parent #5)

Parents also expressed frustration with the need to repeat information to different team members during one or across multiple admissions and the fact that providers sometimes lacked knowledge that was already documented in the medical record. Similar to parents, AYAs described their responsibility for educating providers about their medical history and unique aspects of their care. One AYA highlighted the importance of this role given the risk for errors due to incomplete knowledge about their condition. Multiple AYAs described frustration with the medication reconciliation process during hospital admissions:

“*… the med rec is ridiculous, and it’s exhausting … I’ve had times where they’re keeping you up until 2:00 or 3:00 in the morning because they’re having to put your meds in, and I’m like that’s not good for my health anymore because I can’t be coherent enough to be sure that I’m telling you the right medication information. And also, I’m now having health issues because I’m not sleeping*”.

(AYA #5)

***Learner and Researcher*:** Parents described their journey of becoming experts on their child’s illness. This involved researching diagnoses and medications, and learning to understand medical language and how to provide medical care to their child at home (e.g., use of ventilators or urinary catheterization). Parents also worked with medical teams and child life specialists to learn how to talk to their child or other family members about their child’s disease. One parent shared the following:

“*I didn’t know nothing when I first started … and now I know him … I know everything it takes to keep him going and keep him home … Just study it. You can—if you listen to them, they tell you everything. You’ve just got to pay attention and try to absorb as much as you can*”.

(Parent #1)

Similarly, one AYA expressed a desire to be informed about their medical care, asking questions frequently to understand changes in their condition, lab work, and the rationale behind treatment decisions.

***Planner*:** Parents discussed the importance of understanding both short and long-term timelines for their child. They articulated a desire for honesty from medical teams around anticipated lengths of stay during admissions and recovery timelines following procedures. They described the importance of having contingency plans for home to prevent future hospitalizations. Parents also discussed this role in the context of advance care planning, and expressed frustration with prognostic uncertainty. One parent highlighted the stressful nature of the planner role, balancing other responsibilities with the demands of their child’s illness:

“*I’m a planner. I like to know what’s coming, what’s ahead of me … I have to go to work and we have to plan for family. We have to plan to pay our mortgage. We have to plan all these things*”.

(Parent #6)

Similar to parents, AYAs discussed the importance of contingency planning. One AYA described functioning in a care-coordination role to ensure their discharge needs were met after hospitalizations. AYAs also aligned with parents in their desire to know what to expect with their medical care, appreciating honest timelines and expectations from their providers:

“*There was a surgeon that did my stomach surgery, and any question I asked of him he told me exactly as it was going to be. I appreciated that beyond words because you know what you’re getting into, you can expect what is going to happen … Tell me—be honest with me and tell me how it’s going to be*”.

(AYA #7)

- Social-Emotional roles (Table 3):

***Worrier*:** Parents expressed worry about their child’s disease progression, potential suffering, and eventual death. They feared making the wrong decisions for their child and worried about the consequences of those decisions:

“*You’re terrified that your child may or may not survive. You’re terrified that you may have made the wrong decision and brought them to the wrong place or that maybe you waited too long or not long enough …*”

(Parent #4)

One parent expressed fears about no longer being able to care for their child. Parents also described anxiety around loss of control and worries about their child’s safety during hospital admissions. One AYA similarly described worry about their own safety during hospitalizations, particularly when hearing errors during the medication reconciliation process. AYAs also described anxiety related to their illness, worries about illness progression, and complications of procedures:

“*I know that I have a lot of anxieties in general surrounding my health because of when your health is out of control, you just get more and more anxieties about what’s gonna happen and how things are gonna work. And how’s that gonna work out? And what do I do if this happens?*”

(AYA #5)

***Source of strength, support, and hope*:** Parents viewed themselves as a source of support and positivity for their child.

“*He’s a survivor. So I’ve never given up on him and long as he wants to keep fighting, I’m going to fight with him*”.

(Parent #1)

One parent discussed their desire to avoid letting their child see them nervous or scared. Another described their role in supporting their seriously ill child’s decisions about their own medical care. The parent and AYA dyad described the transition of this role to the AYA as they became older.

“*So when she was younger, I mean, I always felt I was kind of a cheerleader and turning things around and making it positive, right?*”

(Parent #1)

To that question, the AYA responded,

“*Now I’m doing that to you*”.

(AYA #1)

***Guardian (unique to parents)*:** Parents described protecting their child from pain and discomfort, and from hearing distressing information. They described providing unconditional love and support, and their role to make each day as good as it can be. Some highlighted the importance of setting attainable goals for their child. Parents described the challenge of relinquishing control to hospital staff, while wanting to keep their child safe during admissions:

“*As a parent, we try to control the situations you put your kids in … But when you go to a hospital, you turn over the keys to your child and you just hope that they will do everything in their power to make sure that your child comes back the way that they were handed off*”.

(Parent #4)

- Action-oriented roles (Table 4):

***Advocate****:* Parents described their advocacy role in the context of multiple facets of their child’s care. Some discussed fiercely advocating for their child’s comfort and safety in the hospital. Others described advocating for particular medical treatments based on their unparalleled knowledge of their child’s medical history.

“*I do end up getting doctors that say are you asking or telling me? I’m telling you. Because I don’t have time to waste here and I’m talking about my child’s life*”.

(Parent #3)

Parents described advocating for shared decision-making, team meetings, and the inclusion of primary subspecialists in their child’s care. Some also advocated for their child to receive care in the optimal settings, including at home, or on particular hospital units or ICUs. One parent described advocating for their teen’s voice to be heard and respected by the medical team. Similarly, AYAs described advocating for their own voices to be respected and to be involved in medical decisions:

“*I’ve never known that patients and their families can make a decision, like kind of fight for what they want and their needs are … I never realized that you can adapt and tell them that, oh, you want this and you don’t want that*”.

(AYA #6)

One AYA described the challenge of balancing self-advocacy while working collaboratively with the medical team. Some AYAs expressed the importance of having other advocates (e.g., family members) at the bedside to help when things became overwhelming. For AYAs, this role also encompassed advocating for their own medical needs, including ensuring they had the equipment, medications, and services in place to feel comfortable caring for themselves at home.

***Decision-maker*:** Parents described the immense responsibility of making decisions about their child’s treatments, including life-sustaining interventions, as well as advance care planning. One parent reflected the following:

“*You deal with a child’s illness, but now you’re dealing with a decision that’s going to change the structure of how she lives for the rest of her life, and who am I to make that decision?*”

(Parent #2)

AYAs described becoming primary decision-makers for their own medical care as they transitioned into adulthood. Some AYAs described frustration about decisions being made without their input and expressed appreciation for providers that recognize them as part of the team. AYAs highlighted the existential distress and emotional weight of making decisions about their own resuscitation status. They expressed frustration with the need to communicate these difficult decisions repeatedly, and to unfamiliar providers.

“*To have a stranger coming in and asking you randomly, oh, do you feel like dying today? If you want to stay dead if you code on the table during this procedure, and just a random stranger asking that*”.

(AYA #5)

Both parents and AYAs emphasized their desire to receive guidance from medical teams to help with difficult decisions and their appreciation for honest recommendations.

***Communicator*:** Parents described their role as primary communicators with their child, family, and medical teams. Some parents described serving as the voice for their child who could not communicate; others acted as the gatekeeper to determine how much information was shared directly with their child by the medical teams. Parents discussed the responsibility for having difficult conversations with their child about their illness and prognosis. One parent emphasized the unique challenge of communicating with their child about end-of-life:

“*What’s different this time is that the conversation includes him dying, and that is not a conversation I’ve had with him before, and it’s not a conversation I feel comfortable with*”.

(Parent #5)

Similarly, AYAs described becoming the primary communicator with medical teams, highlighting their role in communicating their own goals and values. Some expressed the importance of their voice being heard as adult patients and having a “level playing field” with their doctors.

***Medical care provider (unique to parents)*:** Parents described their role as healthcare providers for their child. For some, this entailed a myriad of daily tasks, including respiratory care, skin care, and medication administration, among others. One parent described their experience learning how to perform urinary catheterization:

“*They’d say you have to cath him...six hours apart four times a day. So I would wake up in the middle of the night and cath him because I was told to*”.

(Parent #6)

***Medical care manager (unique to AYAs)*:** AYAs described taking responsibility for multiple aspects of their own medical care. This included staying informed about their medical condition, managing medications, and ensuring that they had the resources they needed to care for themselves. One AYA emphasized their willingness to do “the work” needed to manage their health and maintain balance with other aspects of their life. AYAs emphasized wanting to be able to care for themselves independently at home, but as one AYA described,

“*You’re the nurse now, you are the doctor now, you are the one that has to make sure all this goes smooth, and it can be overwhelming, for sure*”.

(AYA #7)

## 4. Discussion

The objective of this qualitative study was to explore the experiences of AYAs with CCCs and parents of children with CCCs to conceptualize the breadth of roles each group takes on when engaging with the healthcare system. Our findings demonstrated that both groups share several informational, social-emotional, and action-oriented roles. Only parents described their role as guardians and medical care providers, and the role of medical care manager was unique to AYAs. To our knowledge, this is the first study to explore and compare the self-described roles of these groups within the healthcare system.

Our findings support prior research which has described some of the roles that parents of children with medical complexity and chronic critical illness assume in navigating the healthcare system. Prior research has explored the informational (teacher, learner, planner) and action-oriented (advocate, guardian, decision-maker, communicator, medical care provider) roles parents balance while caring for their seriously ill child [5,6,7,8,27]. The social-emotional roles (worrier and source of strength, support, and hope) have been less extensively studied. Bogetz and colleagues, for example, explored the experiences of parents caring for AYAs with advanced cancer. Parents highlighted their role as supporters and the ways they emotionally “held their child” throughout their cancer journey, while navigating worry amidst uncertainty about their child’s future [28].

Compared with parents of children with CCC, AYAs in our study similarly navigated a complex set of roles, with the added responsibility of managing their own medical care. Much of the existing research exploring AYA roles within the healthcare system has focused on the period of healthcare transition from pediatric to adult care. Studies have explored adolescent experiences and preferences within the roles of communicator, planner, decision-maker, advocate, and medical care manager in the cancer, heart disease, cystic fibrosis, diabetes, CKD, and other serious illness populations [9,10,13,15,16,17,29,30,31,32,33,34]. The majority of existing research describing the social-emotional roles (source of strength, support, and hope and worrier) has focused on AYAs with cancer. Bennett and colleagues found that hope was described as “a source of mental sustenance” for AYAs with advanced cancer during their treatment [35]. In a study by Rosenberg and colleagues, AYAs with recently diagnosed cancer described personal strength and positivity as contributors to resilience, while overwhelming stress, worries and fears were inhibitory factors of resilience [36]. Our study adds to this body of research by examining these social-emotional roles in a group of AYAs with diverse CCCs.

While there was significant overlap in the self-described roles of parents and AYAs, our study reveals important differences in how the groups experience these shared roles. For instance, both parents and AYAs identified as primary communicators with medical providers. However, parents also described the additional responsibility of being their child’s voice and facing the unique challenge of discussing illness, including end-of-life conversations with their child. Decision-making roles also involved distinct stressors for AYAs and parents. AYAs reported existential distress related to decisions about resuscitation status or end-of-life care, whereas parents felt immense anxiety about making life-altering choices for their child, often questioning their qualifications and fearing regret. Additionally, AYAs expressed particular challenges in the teacher role, where they needed to educate healthcare providers while managing the physical toll of their illness. These nuanced differences highlight the need for clinicians to tailor their support to each group’s unique experience.

The overlap of roles among parents and AYAs raises the question of how some of these roles may shift as children grow into adolescence and young adulthood. Previous research has shown that the transition of roles between parents and AYAs is dynamic and evolves over time depending on multiple factors [12]. As children transition into adolescence and young adulthood, it is prudent that medical providers inquire about their preferences for providing and receiving information, communication with medical teams, and decision-making. Research exploring the decision-making preferences of AYAs with serious illness has shown that some AYAs may continue to appreciate more intensive support from parents/caregivers while others may appreciate more autonomy and independence [10,15,17,29,31]. Likewise, parental preferences for the engagement of their seriously ill child or AYA within some of these shared roles will evolve over time, and these preferences require iterative reassessment. Multiple studies have highlighted the need for additional interventions to better meet both parent and AYA needs in the decision-maker role and beyond [29,30].

We recognize that roles such as advocate, communicator, decision-maker, and guardian may intersect in their descriptions. However, this overlap reflects the reality of how adolescents and parents conceptualize their roles in the context of healthcare. Roles are inherently fluid and interdependent rather than neatly siloed. Adolescents and parents often assume multiple, shifting responsibilities based on context, illness severity, and developmental stage. For example, a parent acting as a guardian may simultaneously serve as an advocate when ensuring their child’s needs are met in a medical setting. An adolescent who identifies as a communicator may also take on elements of decision-making when engaging with healthcare providers about their preferences. Parents and AYAs may assume both advocacy and communicator roles when interacting with medical teams to fight for their needs, while the communicator role also encompasses the responsibility of speaking with a child or other family members about serious illness. The overlap in role descriptions reflects the complexity of these interactions rather than a lack of clarity in our framework. While previous research has often categorized these roles as distinct, our findings suggest a more nuanced, dynamic model where roles blend depending on situational demands. This contributes to the literature by challenging rigid role classifications and encouraging a more flexible understanding of adolescent–parent interactions in healthcare communication and decision-making. We acknowledge that our study did not fully explore the nuanced intersections between these roles. Future research could further delineate the conditions under which these roles shift, how different stakeholders perceive role boundaries, and how external factors (e.g., clinician interactions, healthcare policies) influence these dynamics.

While this study expands upon our understanding of the healthcare experiences of AYAs and parents navigating CCCs, there are several important limitations. Participants were recruited from only two academic centers, with a relatively small and racially/ethnically homogenous sample. Participants were all English speakers due to the study team’s language abilities. Each of these factors may reduce the generalizability of the findings, and future studies should aim to capture the perspectives of a larger, more diverse group of participants. Additionally, the study focused on self-described roles within the medical system and specific to serious illness. This overlooks the broader range of roles and responsibilities parents and AYAs manage outside of healthcare, as well as the impacts of sociocultural and economic factors that influence these roles. Further research exploring roles of parents and AYAs both within and beyond the medical system is needed to better understand the full scope of their experiences. Additionally, recognizing this study was a secondary analysis, we did not have the opportunity to explore parent and AYA perceptions of how their roles evolved and in what time-frame. Future research should explore the longitudinal evolution of these roles. Finally, there was a substantial gap between the time of data collection and the time of publication, related to several factors, including the COVID-19 pandemic. While acknowledging this delay, we believe that, given the consistency of our findings with more recently published literature [4,12,16,28,37], the experiences and roles described remain relevant in our current healthcare context.

Despite its limitations, our study underscores important insights for enhancing support for AYAs with CCCs and parents of children managing these conditions. Understanding the multidimensional, evolving roles these individuals hold can help healthcare providers optimize care experiences. Parents of seriously ill children have identified clinical strategies to aid in their medical journey [3,37], while research on AYAs emphasizes the challenges of transitioning from pediatric to adult care and potential strategies for improvement [9,14,30,32,38,39]. This study helps to inform additional strategies to ease the burden of and/or support some of the parent and AYA roles, which include, for example:

- **Teacher role**: Enhancing continuity of care can reduce the need for re-educating new providers [7,37]. For example, some pediatric ICUs have adopted continuity attending and nursing teams for long-stay patients, a strategy that can enhance informational, management, and relational continuity of care for patients and families [40].
- **Decision-maker role**: Goal-concordant recommendations from medical providers may mitigate isolation and decisional regret [41,42]. Too often, when faced with complex medical decisions, clinicians offer patients and families a menu of options and ask them what they want to do. As many of the participants in this study articulated, they appreciate guidance from their trusted providers when difficult decisions arise. Porter and colleagues proposed a useful framework for eliciting a family’s goals of care in the context of their current prognostic understanding, and translating those goals into appropriate medical recommendations [41].
- **Communicator role**: Child life specialists and psychosocial providers can guide parents in difficult conversations, including end-of-life discussions with patients and other family members, including siblings [42,43,44]. The Courageous Parents Network, an exceptional resource for parents of children with serious illness, also has specific guides to support parents navigating difficult conversations about prognosis with their child and siblings [45].
- **Planner role**: Basu and colleagues described strategies for overcoming perceived patient and family barriers to advance care planning. These included normalizing ACP, starting ACP conversations early in a child’s illness trajectory and continuing them longitudinally, and improving clinician communication training [46]. Communication programs and tools have been developed to support clinicians navigating these conversations with parents and AYAs [20,47].

We should continue to strive to better support patients and families within their multidimensional roles. We also must acknowledge the impact that balancing these complex roles while navigating serious illness can have on parent and AYA well-being. Both AYAs with CCCs and parents of children with CCCs experience high rates of depression and anxiety, in addition to other adverse health outcomes [48,49,50,51,52]. Families have identified the importance of peer connection and support, which has also been found to be associated with decreased rates of anxiety [53]. Recognizing the complexity and burden of these roles, healthcare providers should regularly assess coping mechanisms, screen for mental health concerns, and offer emotional and psychosocial support [3,54,55,56,57,58,59].

## 5. Conclusions

This study contributes to a growing body of research aimed at better understanding the experiences of AYAs and parents navigating CCCs. We found that both AYAs with CCCs and parents of children with CCCs navigate similarly complex roles within the medical system, though their experiences can differ and present unique challenges. Understanding these multidimensional roles is crucial for healthcare providers to better support patients and families, especially during the transition to adolescence and young adulthood. Providers should engage in regular, open communication with adolescents and parents to explore preferences and acknowledge shifting roles. Recognizing the intensity and burden of these roles, healthcare providers should routinely assess coping, screen for mental health concerns, and offer emotional and psychosocial support to AYAs and parents of children navigating serious illness. Future research should expand on the roles that AYAs and parents assume outside of the healthcare context, and evaluate how these roles shift and evolve over time. Through this, we can continue improving our understanding of the experiences of AYAs and parents, ultimately enhancing their care and well-being in the healthcare system.

## Figures and Tables

**Table 1:** Parent and AYA Demographics.

Participant Characteristics	Parents (N = 9)	AYAs (N = 7)
Parent age in years, mean (median, range)	52 (52, 28–66)	
Child age in years, mean (median, range)	18 (20, 6–26)	24 (25, 17–32)
Male sex, *n* (%)	3 (33)	2 (29)
Duration of illness in years, mean (median, range)	16 (18, 4–26)	22 (20, 12–32)
Race, *n* (%)		
White	8 (89)	6 (86)
African American	1 (11)	
Asian American		1 (14)
Religion, *n* (%)		
Christian	6 (67)	2 (29)
Jewish	1 (11)	
No religious preference	2 (22)	4 (57)
Disease category, *n* (%)		
Congenital and chromosomal	3 (33)	1 (14)
CNS static encephalopathy	2 (22)	0
CNS progressive	1 (11)	0
Cardiac	1 (11)	1 (14)
Oncologic	1 (11)	1 (14)
Pulmonary	1 (11)	3 (43)
Renal	0	1 (14)
Pediatric Palliative Care involvement, *n* (%)		
Yes	7 (78)	5 (71)
Advance directive, *n* (%)		
Yes	5 (56)	3 (43)
Child or AYA Deceased, *n* (%)	3 (33)	4 (57)
Insurance Type, *n* (%)		
Public	3 (33)	5 (71)
Combined	5 (56)	1 (14)

**Table 2:** Exemplar quotes from AYAs and parents about their self-described informational roles.

Themes	Parent/AYA Quotes
***Informational Roles***	
Teacher	“*And when I’m in the ED, I feel like I spend hours explaining and teaching—which I get, it’s a teaching hospital—but really, I just need to get up to a floor and get him cared for… [NAME] is a unique and complex case, so sometimes I feel like I’m kind of running in circles*”. (Parent #8) “*I’m trying to educate them but I’m trying to treat him and there’s protocol that is a textbook protocol and then there’s protocol that’s…that’s your child’s protocol, and a lot of times those are two very different things*”. (Parent #8) “*Our medical history is super important because decisions can get made in the best of intentions without knowing that there’s some pretty drastic differences that operate within me … we’re not run-of-the-mill patients*”. (AYA #7)
Learner/Researcher	“*But when [NAME] first got moved to the ICU, it was terrifying. And we didn’t know what was happening. The medical staff didn’t know what was happening. And it was like a whole new vocabulary that we’d never heard it, and yet it was happening to our child*”. (Parent #5) “*So I try to ask questions, as I have them come into my head. And I like to understand why changes are being made, or what’s going on, what lab work means, and stuff like that*”. (AYA #5)
Planner	“*I tend to be a planner. And it just helps me to know a little bit, like are we looking at three months, three years, three weeks … And I think that’s a protective move on my part so that if some—if his end of life is soon, that I can begin to be prepared for it*”. (Parent #5) “*If I had a dollar for every time my husband and I had the conversation of what can we expect, what’s going to happen, what does [NAME]’s future look like so we can plan—nobody can answer that question*”. (Parent #8) “*I think my goal is always the shortest stay possible with me being able to comfortably go home and do things independently, if that means IV antibiotics at home, but just being able to manage them on my own safely … and then having the right services in place in case I feel like I can’t manage that on my own, who I can call, and just a lot of that*”. (AYA #5)

**Table 3:** Exemplar quotes from AYAs and parents about their self-described social-emotional roles.

Themes	Parent/AYA Quotes
***Social-emotional roles***	
Worrier	“*The emotional recovery of going through something like this is going to be easily as traumatic as the physical recovery … It’s going to take us years to be able to sleep at night and not worry about him dying in his sleep*”. (Parent #4) “*I could list a ton of concerns; they’re not really about his illness. I’m concerned that I can’t take care of him. I’m concerned that he’s going to suffer. I’m concerned that he’s going to die before I do so that I won’t be here to take care of him. I’m concerned the seizures are going to get worse*”. (Parent #8) “*With this current exacerbation, thinking that I’ve been trying to push down all of my possible fears … and thoughts and anxieties because I’m afraid that I’m gonna get—start crying and get worked up and not be able to breathe even more*”. (AYA #5)
Source of strength/ support/hope	“*If he sees me getting nervous and—he knows when I’m having a hard time … If they know I’m panicking, then he’s going to panic, so I have to stay calm because he—that way he stays calm and everything works. Even though I’m worrying, I still have to hide it as best as you can*”. (Parent #1) “*I believe that there’s something that is around and that—almost like a karma-driven type of thing of be a good person—be a good person, promote good, give off good and in a perfect world that will come back to you*”. (AYA #7)
Guardian	“*He can’t see, so I try to—I shield him from stuff. I try to steer him towards stuff that he can do, and he can enjoy*”. (Parent #1) “*I think everybody who’s human loves their child unconditionally … When you have a special needs kid … You accept them for where they are. And all you want in life for them is to be happy and to be comfortable … You just want your child to leave here with the ability to do and pursue the things that bring them joy*”. (Parent #4) “*And if there’s nothing we can do, then that gets taken off my list as [NAME]’s mom and protector, and I know that what—the to-do part is, is help him make each day he’s here good—as good as we can*”. (Parent #5)

**Table 4:** Exemplar quotes from AYAs and parents about their self-described action-oriented roles.

Themes	Parent/AYA Quotes
***Action-oriented roles***	
Advocate	“*But I mean, it’s your kid. You should speak up for them. I mean, you’re the best advocate for them. If you see something that you don’t think is right, have the doctor explain it to you*”. (Parent #1) “*It’s really [NAME]’s body and his disease that’s going to set the pace, and that it kind of helps me shift back to yes, and our role as his parents are to advocate for him, to be his voice, to help him have the best days possible when he’s alive*”. (Parent #5) “*I’m an adult. I need to take care of my health, and it’s my body, my health. What do I want? What are my needs? And let me see*”. (AYA #5)
Decision-maker	“*And I think time helped me understand that I don’t have to do everything that some—that the medical team suggests. I’m a part of the team too, and I can make decisions*”. (Parent #6) “*So a lot of [NAME]’s doctors and nurses will say well, it’s up to you. Yeah, I know it’s up to me, but give me some guidance. Give me some guidance. You do this every day*”. (Parent #6) “*But when somebody comes in and asks you, what do you want your code status to be, they just dropped this bomb, you answer, and then they just walk out. And no one comes back later. And it’s like are you okay? … they just leave that on you*”. (AYA #5)
Communicator	“*We’re actually struggling right now with how and when to have the conversation with him that we’ve learned that his lifespan is limited. Right now, we have not talked with him about that yet … I feel uncomfortable with those conversations*”. (Parent #5) “*I did have an issue last admission where people would walk in the room and talk about a specific subject right in front of [NAME], and that subject really shouldn’t be talked about in front of a patient if that patient doesn’t have the capacity to make the decisions on his own … I don’t know how much he understands, and I don’t want him to stress about things that he doesn’t have the capacity of knowing*”. (Parent #6) “*I know what my wishes are, every time somebody comes in… I’m just like I want everything the same. And I just have to have this emotionless, cold answer because I’m like I can’t think about this. I know what my wishes are. And I don’t want to think about it because I’m not gonna flip-flop on it. But it’s a hard conversation to have because—well because they have to bring it up, even if they’re not anticipating anything*”. (AYA #5)
Medical care provider	“*You’ve just got to pay attention and try to absorb as much as you can … I watch what they do up here so when I get home, if I see the same thing happening to me, I can try to do it at home and prevent a hospitalization*”. (Parent #1) “*And then there were times I said to them, get me out of here. I’m ready. And they never used to discharge from ICU until me. And I’m like hey, if she’s good enough to go to the floor, she’s good enough to go home. Because I’m—once you hit the floor, I’m doing all the work anyway*”. (Parent #3)
Medical care manager	“*In this current moment, my health I would consider failing, end-stage* CF. *I’m having enough kidney issues, them failing. My lung function is very low. And there’s not a lot of—any treatment-wise, I’m just maintaining … there’s no possibility of improvement of my lung function. There’s no getting better at this point for—I’m having exacerbations, but there’s no big picture getting better. So it’s definite like I’m end-stage, heading downhill*”. (AYA #5) “*I think they understand pretty well. I think that I’ve voiced it clearly enough times that they echo—have a pretty good understanding of what my goals are, what my value factors lie within and also the element that I don’t mind putting the work in. The work is part of it, and I want my health but I also—I want a life*”. (AYA #7)

## Data Availability

This study includes minor-aged patients and their parents who did not provide consent for all details/data to be shared.

## Abbreviations

The following abbreviations are used in this manuscript:

AYA	Adolescent and young adult
CCC	Complex chronic condition
BCH	Boston Children’s Hospital
ACP	Advance care planning
